# 

**Published:** 1860-04

**Authors:** 


*Books and Pamphlets Received.*

A Medico-Legal Treatise on Malpractice and Medical Evidence, comprising the Elements of Medical Jurisprudence. By John J. Elwell, M.D., Member of the Cleveland Bar. New York: John S. Voorhies. 1860.

Lectures on the Diseases of Infancy and Childhood. By Charles West, M.D., &c. Third American, from the Fourth Revised and Enlarged London Edition. Philadelphia: Blanchard & Lea 1860.

The Diseases of the Ear; their Nature, Diagnosis and Treatment. By Joseph Toynbee, F.R.S., &c., with 100 Engravings on Wood. Philadelphia: Blanchard & Lea. 1860.

Clinical Lectures on Certain Acute Diseases. By Robert Bentley Todd, M.D., F.R.S., &c. Philadelphia: Blanchard & Lea. 1860.

The Action and Sounds of the Heart; a Physiological Essay. By George Britton Halford, M.D., &c. London: John Churchill. 1860.

On the Circulation of the Blood in the Venous System during Life. By George Murray Humphrey, M.D., F.R.S., &c. Cambridge: Macmillan & Co. 1859.

On Excision of the Knee-Joint. By Oliver Pemberton, Surgeon, &c. London: T. Richards. 1859.

